# Porosity Distribution in Apically Perforated Curved Root Canals Filled with Two Different Calcium Silicate Based Materials and Techniques: A Micro-Computed Tomography Study

**DOI:** 10.3390/ma12111729

**Published:** 2019-05-28

**Authors:** Saulius Drukteinis, Vytaute Peciuliene, Hagay Shemesh, Paulius Tusas, Ruta Bendinskaite

**Affiliations:** 1 Institute of Dentistry Faculty of Medicine Vilnius University; Zalgirio 115, LT-08217 Vilnius, Lithuania; vytaute.peciuliene@gmail.com (V.P.); paulius.tusas@gmail.com (P.T.); rutabend@gmail.com (R.B.); 2 Academic Centre for Dentistry Amsterdam (ACTA); Gustav Mahlerlaan 3004, 1081 LA Amsterdam, The Netherlands; H.Shemesh@acta.nl

**Keywords:** apical plug, BioRoot RCS, micro-computed tomography, MTA flow, root perforation, tricalcium silicate

## Abstract

The present study evaluated the porosity distribution of BioRoot RCS/single gutta-percha point (BR/SC) and MTA flow (MF) fillings, which were used as plugs for the apical perforation repair in curved canals of extracted mandibular molars using micro-computed tomography (μCT). Forty mesial root canals of mandibular first molars were shaped with ProTaper NEXT X1–X5 files 2 mm beyond the apex to simulate apical perforations that were randomly divided into two groups (*n* = 20) according to the material and technique used for the apical plug: BR/SC or MF. The specimens were scanned before and after canal filling at an isotropic resolution of 9.9 μm. The volumetric analysis of voids in the apical 5 mm of the fillings was performed. Data were analyzed using one-way ANOVA with Bonferroni correction (*p* < 0.05). Micro-computed tomography (µCT) evaluation revealed significant differences between the groups in terms of porosity: the total volume and percentage volume of voids was lower in the BR/SC group in comparison with the MF group (*p* < 0.05), with the predominance of open pores in both groups. Neither of the materials and/or application techniques were able to produce void-free root fillings in the apical region of artificially perforated curved roots of mandibular molars.

## 1. Introduction

Iatrogenic errors, such as root canal transportations, ledging or zipping, can lead to uncontrolled and accidental apical or lateral root perforations. The risk of perforations significantly increases during root canal retreatment procedures [1,2,3]. Visualization of the perforation site is important and can impact on the success of the treatment; however, direct observation of perforations beyond the curvature of the root canal is limited, even when a dental microscope is used [4]. The lack of direct vision during delivery and condensation of the repair materials leads to a poor apical seal [5,6].

Prior to the introduction of mineral trioxide aggregate (MTA) the success rates of perforation repairs were relatively low because of poor biocompatibility, sealing ability, high cytotoxicity and hydrophobic properties of the materials [7]. MTA has changed existing standards in the management of endodontic complications, vital pulp therapy and regenerative endodontic procedures. However, MTA has drawbacks that made the use of this material challenging for many clinicians. The main clinical challenges are problems with mixing and hardening, long setting time, difficult handling characteristics and complicated delivery of the material, discoloration of tooth tissues and the presence of toxic elements [8,9]. 

During the last decade, hydraulic calcium silicate materials or improved MTA formulations for use as root canal sealers, fillers or root repair materials have been introduced [10,11]. Modifications of the original mineral trioxide aggregate formulation have improved its physicochemical and biological properties whilst facilitating its clinical applicability [12,13]. The new concepts for simplified root canal obturation and management of endodontic complications using flowable hydraulic calcium silicate based materials have been suggested [12,13,14].

BioRoot RCS (Septodont, Saint-Maur-des-Fossés, France) is a hydraulic cement, presented as a powder composed of tricalcium silicate, zirconium oxide and a liquid, which is mainly water-based gel with additions of calcium chloride and a water-soluble polymer [14,15]. It has been proposed that material should only be used with cold canal filling techniques, as the heat generated during thermoplastic obturation can negatively affect the flowability and film thickness of the material [14]. In recent times, the single cone (SC) cold hydraulic obturation technique was suggested for use with hydraulic calcium silicate cements [13,16].

MTA flow (MF, Ultradent Products Inc, South Jordan, UT, USA) is an MTA-based repair material composed of an extremely fine, radiopaque grey powder of di- and tricalcium silicate, which sets with a water-based gel [17]. The material provides superior handling characteristics and variety of consistencies, a more rapid setting time, resistance to wash-out and is the only MTA repair material that can be delivered in the thin consistency using a 29-G needle [18]. It has been shown that novel material possesses the same biocompatibility and bioactivity as the Biodentine (Septodont) and can be used in a wide range of endodontic procedures, including perforation repair [19].

Micro-computed tomography (µCT) as a nondestructive technique is widely used to evaluate the sealability of the filling materials or the quality of root canal obturation and can identify the presence and distribution of the porosity inside fillings and quantify the volume of voids [20,21,22,23,24]. There are several articles reporting the performance of BioRoot RCS as a filling material in conjunction with various root canal filling techniques [22,25]. However, there is no published study evaluating the performance of the BioRoot RCS/single cone fillings as an apical plug for root perforation repair, nor is there data available on the effectiveness and performance of the MF as root canal filling or perforation repair material. The aim of this study was to evaluate and compare the porosity of BioRoot RCS/single gutta-percha point and MF fillings as an apical plug in artificially perforated curved canals of extracted mandibular molars using μCT. 

## 2. Materials and Methods 

### 2.1. Specimen Preparation

After the approval of the local ethics committee (protocol No. EK-2), 20 freshly extracted human mandibular first molars with fully developed roots were selected and stored in a saline solution. All procedures were performed by a single operator—an experienced endodontologist. Endodontic access cavities were prepared using high-speed burs (Endo Access, Dentsply Sirona, Ballaigues, Switzerland) and water-cooling. Preoperative radiographs were taken and the curvatures of the mesial roots were calculated as determined by Schneider’s method [26]. Only molars with a moderate 10°–20° curvature and two separate mesial root canals where a size 10 K-file (Dentsply Sirona) could be inserted to full working length (WL) were selected.

The working length was determined by inserting a size 10 K-file into the mesial root canals until the tip was visible at the apical foramen under 10× magnification (OPMI Pico; Carl Zeiss, Oberkochen, Germany). The length of the file was increased by 2 mm and the instrument was reinserted into the canal 2 mm beyond the apex, simulating over-instrumentation. The glide path was subsequently created with a size 15 and 20 K-Flexofile (Dentsply Sirona). Root canals were enlarged with ProTaper NEXT (Dentsply Sirona) instruments used in rotary movement with a rotation speed of 300 rpm, and a torque of 1 Ncm. The instrumentation sequence was performed using the following instruments: ProTaper Universal SX (Dentsply Sirona) in the coronal and middle thirds of the root canal and X1 (size 17, 0.04 taper), X2 (size 25, 0.06 taper), X3 (size 30, 0.07 taper), X4 (size 40, 0.06 taper) and X5 (size 50, 0.06 taper) 2 mm beyond the apex. Instruments were driven using an 8:1 reduction hand-piece powered by an endodontic motor (X-Smart; Dentsply Sirona). 

The root canals were irrigated with 5 mL 3% sodium hypochlorite after each instrument. At the end of preparation, 5 mL of 18% ethylenediaminetetraacetic acid (Ultradent Products Inc.) was used for 3 min and distilled water was used as a final flush to remove the irrigants from the root canals. Irrigation was performed using disposable syringes and 29-G NaviTip needles (Ultradent Products Inc.). Root canals were dried with paper points. The root tips of the specimens were covered with a polytetrafluoroethylene (PTFE) tape (Tesa SE, Norderstedt, Germany). Teeth were fixed into prefabricated A-silicone (3M Express, 3M ESPE, Seefeld, Germany) blocks up to the cemento-enamel junction to simulate surrounding bone and ensure the blindness of the canal filling procedure. 

### 2.2. Root Canal Obturation

Specimens were numbered and randomly assigned into two groups (10 teeth and 20 mesial root canals per respective group) using a true randomness generator (www.random.org) according to the material and technique used for canal filling: BioRoot RCS/single gutta-percha point (BR/SC) (BioRoot RCS; Septodont) and single gutta-percha cone or MF (Ultradent Products Inc.).

In the BR/SC group, the canals were filled with BioRoot RCS (Septodont) sealer and ProTaper NEXT X5 (Dentsply Sirona) gutta-percha points using a single cone cold hydraulic obturation technique. The gutta-percha point was cut 4 mm shorter than the WL with a sterile scalpel and fitted to the root canal with a tug back 2 mm shorter than the perforated apex of the root. The sealer was mixed according to the manufacturer’s instructions and inserted into the back of the clear Skini syringe (Ultradent Products Inc.). The plunger was reinserted into the syringe and the plastic cannula (Capillary Tip; Ultradent Products Inc.) was adjusted. The sealer was injected into the root canal approximately 2 mm shorter than the determined WL, gently pressing the plunger of the syringe and withdrawing the cannula until the sealer was visible at the orifice of the root canal. The pre-fitted gutta-percha point was coated with a small amount of the sealer and inserted into canal 2 mm shorter than the WL. 

In the MF group, the root canals were filled with MF mixed according to the manufacturer’s recommendations. A total of 0.19 g of the powder was mixed with 3 drops of the liquid and the thin consistency of the cement was obtained. After mixing, the material was inserted into the back of the clear Skini syringe, the plunger was reattached and a 29-G NaviTip needle (Ultradent Products Inc.) was fitted. The small amount of the MF was expressed through the tip and syringe with a NaviTip (Ultradent Products Inc.) was inserted into root canal 2 mm from WL. The material was delivered to the canal by gently pressing the plunger of the syringe and withdrawing the needle until the MF was visible at the orifice of the root canal.

Postoperative radiographs were taken immediately after root canal filling to confirm the length and homogeneity of the root canal fillings. If the voids inside the filling or inadequate length of the root filling were detected, the root filling procedure was repeated by additionally injecting MF or BioRoot RCS (Septodont) and reinserting a gutta-percha point to the previous length. New post-filling radiographs were taken to confirm their quality. After the setting of the BioRoot RCS (Septodont), the gutta-percha cone was cut with a heat carrier. The endodontic access cavities were filled with Cavit W (3M ESPE, Seefeld, Germany) and all specimens were stored in a saline solution at 37 °C for 1 week to allow the filling materials to set completely. 

### 2.3. µCT Scans and Analysis

The specimens were scanned before and after the root canal filling using a high-resolution µCT scanner (SkyScan 1272; Bruker-microCT, Kontich, Belgium). All scans were performed at 100 kV, 100 mA and an isotropic resolution of 9.9 μm. A 0.11-mm copper filter was used for artefact reduction and each projection was obtained using 360° rotation, with a camera exposure time of 1073 ms and a rotation step of 0.4. Images of the specimens were reconstructed using a ring artefact reduction factor of 6 and beam hardening correction of 20% with NRecon v. 1.6.9.18 software (Bruker-microCT).

The CTAn v.1.14.4.1 software (Bruker microCT) was used for quantification of the root canal volume (CVol) and filling material volume (FVol). The gray scale range required to recognize each object was determined in a density histogram by using a global threshold method. Comparisons between the original and segmented scans were performed to ensure segmentation accuracy. Task lists based on arithmetic and logical operations were applied to create separate binary images of the root canal and the filling material by using a custom-processing tool. 

The volume of voids (VVol) was determined by subtracting the filling material volume from the pre-obturation root canal volume: VVol = CVol − FVol.

The percentage volume of voids (%VVol) was calculated by using the following formula: %VVol = VVol × 100/CVol. 

The volume of the open and the closed pores was calculated using CTAn v.1.14.4.1 software’s (Bruker microCT) Bitwise operations function. The percentage volume of open pores (%OPVol) and closed pores (%CPVol) was calculated by using the following formulas:

%OPVol = VVol × 100/OPVol(1)

%CPVol = VVol × 100/CPVol(2)

All analyses were conducted for a volume of interest (VOI) of 5 mm. The CTVol 1.10.1.0 software (Bruker microCT) was used for 3D volumetric visualization and qualitative evaluation of the fillings.

### 2.4. Statistical Analysis

The Shapiro–Wilk test was used to confirm the normality of the data. The differences between the groups were compared using one-way analysis of variance (ANOVA) followed by Bonferroni correction. The significance level was set at 5%. These analyzes were performed using SPSS 25.0 software (SPSS Inc., Chicago, IL, USA).

## 3. Results

**Figure 1:**
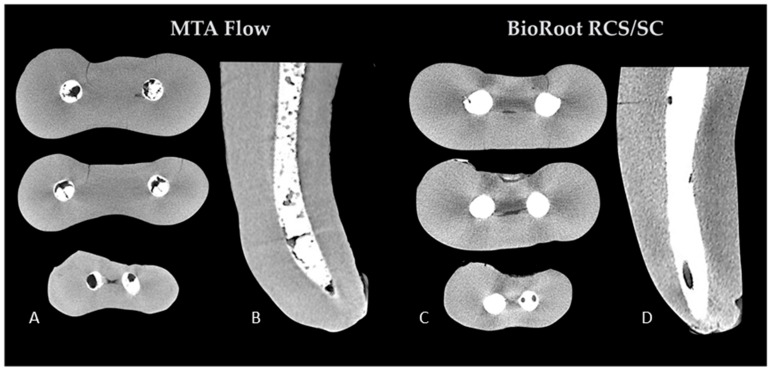
Representative cross-sectional images at the 5 mm, 3 mm and 1 mm (top to bottom) levels of the volume of interest (**A**,**C**) and two-dimensional (2D) sections (**B**,**D**) of the mesial roots of random samples of two experimental groups, demonstrating the porosity of the fillings. MTA: mineral trioxide aggregate.

The µCT evaluation revealed pores of various sizes inside the filling materials as well as in the interface of the fillings and root canal walls in both groups (Figure 1).

The results of the volumetric analysis of the fillings in both groups are shown in Table 1. There were no significant differences in the root canal volume before the root filling procedures (*p* > 0.05), indicating no significant volumetric variances between the prepared root canals of the specimens in two experimental groups. However, the mean volume of voids and the percentage volume of the voids were significantly different between groups (*p* < 0.05). MF fillings had more pores when compared to BR/SC. 

The results of the porosity distribution in the fillings in both groups are summarized in Table 2. The mean volume of the open and the closed pores was significantly different between groups (*p* < 0.05). MF fillings had a higher volume of open and closed pores due to the overall higher porosity of the material when compared with BR/SC. However, there were no significant differences in the percentage volumes of open and closed pores (*p* > 0.05), demonstrating that the majority of pores were distributed at the interface of the fillings and root canal walls in both groups. 

**Figure 2:**
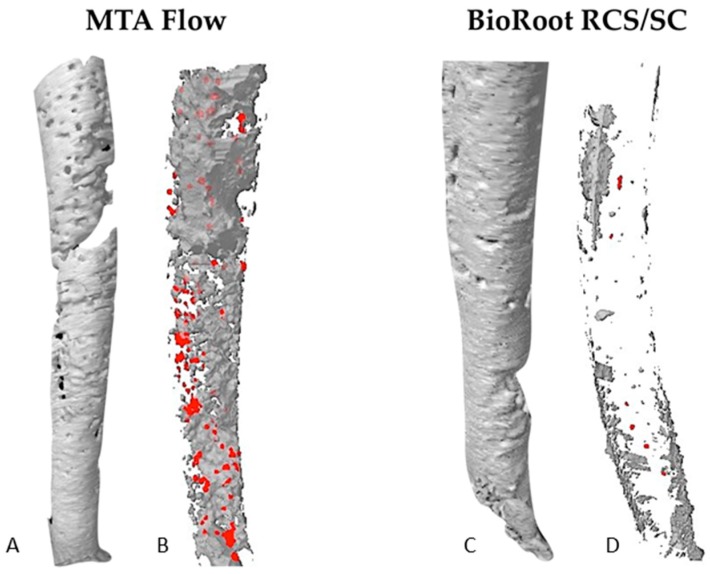
The three-dimensional (3D) reconstructions of the fillings of the random samples demonstrating the different size and distribution of the voids through the filling materials (**A**,**C**). 3D reconstructions of the open (grey) and the closed (red) pores inside the filling materials (**B**,**D**).

The representative three-dimensional (3D) images of the open/closed pores’ distribution of the specimens in both groups are illustrated in Figure 2.

## 4. Discussion

One of the iatrogenic complications that can occur during root canal treatment or retreatment procedures is a root perforation. It has been shown that 53% of all perforations occur during prosthetic and 47% during endodontic treatment procedures [8]. The seal of a perforation site is a key factor in their successful management. It has been concluded that “experience of treatment providers” and the use of biocompatible materials such as MTA are important prognostic factors for successful perforation repair and superior healing rates [3,4,27]. Gorni et al. [4] have found that perforations in molars had less healing potential, and the progression of the inflammatory process was more likely to occur in patients with apical or middle compared with coronal perforations. The differences in success rates could be related to the lack of visualization under magnification in molars during the sealing of perforation in curved roots [4]. Nevertheless, it was also shown that a lack of knowledge in the use of these materials exists. Ha et al. [6] demonstrated that almost all endodontists (98.8%) and only 39.8% of general practitioners used MTA for perforation repairs. The main reasons for such rare use of MTA among dentists could be the lack of experience in the handling of the material rather than its cost [28]. These findings indicate that the use of MTA is a significant clinical problem and that new materials and simplified techniques for perforation repair are needed.

To simplify root canal filling, premixed or powder/liquid formulations of flowable hydraulic calcium silicate-based materials were introduced, using various delivery methods as well as new filling techniques [12,22]. It has been shown that BioRoot RCS can be successfully used as a sealer as a biologic filler in conjunction with a solid cone to create hydraulic pressure inside the root canal [14]. However, until now there was no data on the characterization of the MF as a root canal filler. Therefore, the performance of these fillers as perforation repair materials has not yet been investigated. It has been shown that both materials tested possess all the biological properties of original MTA but with more advantageous physical and handling properties [13,19,22]. The biocompatibility and bioactivity of the perforation repair material, as well as its dimensional stability over a long period of time, are crucial for the hermetical seal of the perforation site and better success rates of perforation repair [4,22]. The current study was specifically designed to represent real clinical conditions in the case of the management of apical perforations in mandibular molars using these novel materials and techniques. The proposed concept relies on the use of flowable materials as injectable biological fillers, therefore, in the present study, BR and MF as injectable biological fillers were applied to the apical perforations. The aim of this study was to characterize the two different materials placed as apical plugs using two different techniques, in order to achieve the same clinical result—the tight apical seal of the perforated root. This simplified technique can be clinically appealing because it does not require superior handling skills or the direct visual control of the procedure. 

It has been demonstrated, that no filling material or technique can ensure void-free three-dimensional root canal fillings [12,22]. The evaluation of the porosity of the fillings is widely used to assess the sealability of the materials and the quality of obturation [20,21]. However, it should be mentioned that the key issue in terms of porosity is whether the voids are through-and-through (open pores) and allow leakage, or simply internal voids (closed pores) with no impact [21,22,23]. Previous studies have demonstrated porosity of MTA when placed as an apical plug, retrofilling or root canal filling material, and no technique used for compaction of MTA produced void-free obturation [24,29]. In this study, the µCT evaluation revealed that the overall percentage volume of voids of MF reached 19.06% and was significantly higher in comparison to BR/SC fillings (3.45%) or previously assessed different commercially available MTA formulations [24,29]. The results of the present study are in concordance with previous findings, demonstrating that the porosity of hydraulic calcium silicate-based cements used in conjunction with a single gutta-percha point is comparable to conventional root fillings and techniques [12,13,22] or even superior [14,20]. The distribution of the voids in the fillings was similar in both groups: the majority of the voids were classified as “open pores” (80% in the BR and 84.7% in the MF group) and just 20% and 15.3% as “closed pores”. However, because of the higher overall porosity of the MF, the volume of open pores was significantly higher in comparison with BR/SC fillings. The volumetric visualization revealed the “network” of the through-and-through open pores in the MF fillings, while the open pores in the BR group were much more isolated. However, the porosity of MF was not previously evaluated, thus it was not possible to compare the present findings with the results of previous studies. It could be that the high amount of porosity in MF is related to the amount of liquid added to make an extremely flowable paste and entrapment of air bubbles during the mixing procedure [30,31,32]. It should be mentioned that the present study evaluated the porosity distribution in the apical 5 mm of the perforated root canals, filled using different materials and techniques. These differences could influence the higher overall porosity of MF fillings in comparison with BR/SC, as the solid gutta-percha point was used to create the hydraulic pressure inside the root canals obturated with BR. If BR were a very porous material, a much higher volume and the percentage of the overall porosity would be detected. However, our results indicated the opposite.

µCT was widely used in endodontic research, however, no studies have evaluated the porosity of flowable bioceramic materials used as perforation repair materials in artificially perforated curved roots of mandibular molars [12,22,33,34]. Despite the advantages of µCT, there are some risks of mistakes and difficulties determining the differences between the two objects of different densities (filling materials vs. the root canal walls). Zaslansky et al. [35] reported that even the best µCT images could have misleading differences in the contrast between the two different interfaces. In the current study, to minimize these human errors and differences, the pre-obturation image sets were used to evaluate the root canal volume while the volume of the fillings was calculated from post-obturation data sets. It should be mentioned that the 5 apical mm were selected as VOI for volumetric analysis in the current study, as fillings were tested as apical plugs and not as a root canal fillings. In the majority of studies, the apical, middle and coronal thirds of root canals were selected as VOI for evaluation, therefore, the absolute values of the present findings may be different because of the various VOIs used for volumetric analyses [5,23,24].

It has been concluded that void-free root fillings are associated with higher success rates in primary root canal treatment and retreatment because of the leakage after root canal obturation [1,2,12]. However, there are only a few reports demonstrating some correlations between the porosity of perforation repair materials and the success rates of the management of the iatrogenic perforations [3,8]. Hypothetically, the high amount of porosity, especially the open pores, in MF can be related to the higher rates of the leakage. Meanwhile, it has been shown that the porosity of the hydraulic calcium silicate-based materials reduces with time in the presence of the liquids [11,12]. Therefore, further studies are needed to investigate if the porosity of the materials tested, especially whether a thin consistency of MF provides an impact on clinical outcomes when used in vivo.

## 5. Conclusions

Within the limitations of this in vitro study, it can be concluded that no materials or application techniques were able to produce void-free fillings placed as apical plugs in artificially perforated curved roots of mandibular molars. However, MF, in its thin mixture, had significantly more pores in comparison with BR/SC fillings, while open pores were predominant over closed pores in both groups. 

## Figures and Tables

**Table 1:** Mean values and standard deviations (SD) of the differences between initial root canal volume, overall volume and percentage volume of voids.

Group	*N*	Root Canal Volume (mm^3^)	Volume of Voids (mm^3^)	Percentage Volume of Voids
BioRoot RCS/single gutta-percha point (BR/SC)	20	2.48 ± 0.51	0.15 ± 0.07 ^a^	3.45 ± 1.71 ^b^
MTA Flow (MF)	20	2.59 ± 0.61	0.72 ± 0.32 ^b^	19.06 ± 9.57 ^b^

Different superscript letters in the same column indicate significant differences between groups (*p* < 0.05).

**Table 2:** Mean values and standard deviations (SD) of the differences between volume and percentage volume of open and closed pores.

Group	*N*	Volume of Open Pores (mm^3^)	Volume of Closed Pores (mm^3^)	Percentage Volume of Open Pores	Percentage Volume of Closed Pores
BR/SC	20	0.12 ± 0.05 ^a^	0.03 ± 0.01 ^a^	80.0 ± 1.71	20.0 ± 1.69
MF	20	0.61 ± 0.12 ^b^	0.11 ± 0.02 ^b^	84.7 ± 8.47	15.3 ± 6.48

Different superscript letters in the same column indicate significant differences between groups (*p* < 0.05).
